# Technological and Safety Characterization of *Kocuria rhizophila* Isolates From Traditional Ethnic Dry-Cured Ham of Nuodeng, Southwest China

**DOI:** 10.3389/fmicb.2021.761019

**Published:** 2021-11-15

**Authors:** Qiao Shi, Xinrui Wang, Zijing Ju, Biqin Liu, Changwei Lei, Hongning Wang, Hong Li

**Affiliations:** ^1^Institute of Agro-Products Processing, Yunnan Academy of Agricultural Sciences, Kunming, China; ^2^College of Life Sciences, Sichuan University, Chengdu, China

**Keywords:** coagulase–negative cocci, starter culture, nitrate reductase, protease, amino acid decarboxylase

## Abstract

Nuodeng ham is known for its unique processing techniques and flavor. In the present study, proteolytic microorganisms from cured artisanal Nuodeng ham were investigated in order to identify and select potential starter cultures for its faster and safer fermentation. Eight isolates, accounting for 57% of proteolytic microorganisms, were found to be related to *Kocuria rhizophila*. Relevant properties of *K. rhizophila* as potential starter culture were evaluated *in vitro* for the first time. Intra-species diversities were found in phylogenetic and physiological properties of *K. rhizophila* isolates. Nevertheless, desirable attributes, such as halo-tolerance, nitrate reductase and protease activity, as well as the absence of antimicrobial resistance and amino acid decarboxylase activity, were observed in selected isolates. Moreover, genome analysis of isolates K24 and K45 confirmed their lack of typical genes for virulence, antimicrobial resistance and amino acid decarboxylase. *K. rhizophila* may thus represent a novel starter candidate of coagulase-negative cocci group and contribute to color and flavor development of fermented meats.

## Introduction

Dry-cured ham is traditionally made by Bai ethnic people in Nuodeng village, Dali, Yunnan Province, thanks to local salt reserves and favorable climate. The making process includes curing with distilled corn liquor before mineral-rich local salt and years of hanging to cure and develop flavor. Nuodeng ham has gained fame nationwide as a traditional and regional specialty. There is a need to identify technologically relevant bacteria from artisanal Nuodeng ham for the selection of starter cultures because autochthonous starters are better adapted to the specific habitat and can help maintain its organoleptic characteristics in a shorter ripening time (Casquete et al., 2011, 2012). Moreover, the competitiveness of autochthonous starters over undesired bacteria could reduce biogenic amine contents in artisanal meat fermented products (Latorre-Moratalla et al., 2012).

Coagulase-negative cocci (CNC) or Gram-positive catalase-positive cocci are important microbial group in meat fermentation. They participate in color and flavor development of dry-cured meats through their enzymatic activities (Fonseca et al., 2013; Vermassen et al., 2016; Cruxen et al., 2017; Hu et al., 2018). Their nitrate reductase activity is responsible for typical stabilized cured meat color *via* the formation of nitrosomyoglobin. Nitrate reductase and catalase activity of CNC also offer protection against severe lipid and protein oxidation, which lead to the deterioration of the color, texture, flavor, and nutritive value of meat products (Sanchez Mainar et al., 2017). Although proteins and lipids in meat tissues are degraded mainly by endogenous enzymes during ripening, the proteolytic, lipolytic and other metabolic activities of CNC are also needed for the generation of the characteristic flavor of meat products (Flores and Toldrá, 2011).

Coagulase-negative cocci mainly comprise coagulase-negative *Staphylococcus* spp. and *Kocuria* spp., with the former belonging to family *Staphylococcaceae* and the latter *Micrococcaceae* (Martin et al., 2006; Talon et al., 2007; Aquilanti et al., 2016). CNC are naturally present on the raw meat or originate from the environment (Rieder et al., 2012). CNC also constitute the predominant microbiota of salt used to cure ham, of which *Staphylococcus* and *Kocuria* account for 60 and 6%, respectively (Cordero and Zumalacarregui, 2000). Under the selective pressure exerted by production process, CNC dominate bacterial communities throughout the ripening of dry-cured hams of different regions (Landeta et al., 2013; Lin et al., 2020). Coagulase-negative *Staphylococcus* spp., such as *S. xylosus* and *S. equorum*, are the predominant CNC in fermented meats, whereas the prevalence of *Kocuria* spp. is low, with an isolation rate of up to 20% of CNC in naturally fermented meats (Papamanoli et al., 2002; Mauriello et al., 2004; Martin et al., 2007; Iacumin et al., 2012, 2020; Sun et al., 2019).

In meat fermentation, starter cultures, mainly lactic acid bacteria and coagulase-negative staphylococci, are often used to standardize product properties, shorten ripening times, and improve product safety (Alfaia et al., 2018; Laranjo et al., 2019). *K. varians* (formally *Micrococcus varians*) is the most frequently encountered *Kocuria* in meat products and is often used as a starter culture to improve sensorial profile of fermented meats and reduce the generation of biogenic amines (Leuschner and Hammes, 1998; Coloretti et al., 2008; Tremonte et al., 2010). Scannell et al. (2004) selected a *K. varians* isolate with proteolytic activity from dry cure hams and used it in combination with *Lactobacillus sakei* to start a non-dried fermented ham product. The proteolytic *K. varians* was found to influence the amino acid profile thereby potentially enhancing the sensorial attributes of the ham (Scannell et al., 2004). In another study, a mixed starter cultures comprising of *K. varians* and *Lactobacillus acidophilus* brought desirable changes in amino acid profile and sensory properties of wet cured ham (Gogoi et al., 2015). Moreover, the supplementation of *K. varians* as a starter culture with *Lactobacillus plantarum* notably improved the color of salami and decreased its biogenic amines (Coloretti et al., 2008). In spite that members of the genus *Kocuria* constitute an important part of functional CNC in fermented meats, only strains from *K. varians* have been investigated as starter cultures. However, the technological and safety attributes of other *Kocuria* species, e.g., *K. rhizophila*, remain unexamined. *K. rhizophila* has been isolated from fermented seafood (Guan et al., 2011), cheese (El-Baradei et al., 2007), dry-cured ham (Martinez-Onandi et al., 2019), and sausage (Iacumin et al., 2020), and has been associated with typical aromatic traits of naturally fermented sausage (Iacumin et al., 2020).

Traditional Nuodeng ham making involves the application of local salt with distinct mineral profiles on hind leg of local black pig. It is of interest to investigate relevant CNC in the specific microbial ecology shaped by the characteristic production practice and local environment. In order to identify strains that might facilitate Nuodeng ham fermentation, bacterial isolates from native microbiota survived the curing of artisanal ham were firstly selected based on their proteolytic activity. Since a high proportion of isolates (8/14 isolates) with high proteolytic activity were affiliated with *K. rhizophila*, the potential of *K. rhizophila* as eligible starter cultures for fermented meats was explored. To our knowledge, this is the first report on the technological and safety features of *K. rhizophila* for food application.

## Materials and Methods

### Isolation and Screening of Proteolytic Bacteria

Raw hams from local black pig breeds were processed after carcass cooling. Distilled corn liquor of 3% (w/w) was applied on ham surface, followed by salting for three times with a total of 6% (w/w) salt from Nuodeng salt well. The proportion for each time was 3.5, 1.5, and 1%.

After salting for 40 days with a water loss of approximately 15% of ham weight through compression, four hams from two local producers were selected randomly and a slice (about 3 cm thickness) from meat surface of each ham was sampled aseptically. The sample was prepared by subjecting the surface briefly to flame and chopping aseptically the inner part. An amount of 25 g minced meat was then homogenized with 225 g sterile peptone water (1 g/L peptone, 0.85 g/L NaCl, 1 mL/L tween 80) for 2 min in a XY-08 lab paddle blender (Xiyu, Shanghai, China). The supernatant was serial decimal diluted, and 100 μL dilution was spread plated on Plate Count Agar (PCA), Mannitol Salt Agar (MSA) and deMan, Rogosa, and Sharpe (MRS) Agar. After incubated at 30°C for 48 h, 65 colonies on different culture media were isolated. The isolates were then streaked onto litmus milk agar for proteolytic bacteria screening (containing 100 g/L skimmed milk powder, 1.67 g/L litmus, 3 g/L beef extract, 5 g/L NaCl, 2 g/L K_2_HPO_4_, and 15 g/L agar) as described previously with modifications (Molina and Toldra, 1992). Isolates showing large clear zones after incubating were then characterized.

### Taxonomic Identification

The proteolytic isolates were evaluated by Gram staining and catalase activity. For catalase activity, a loop of overnight culture was applied on a slide, followed by adding a drop of 3% hydrogen peroxide. Immediate bubble formation was recorded as positive reaction for catalase activity. *Pediococcus pentosaceus* ATCC 8081 was used as a negative control.

Biochemical characterization was conducted using biochemical panel (HD210033, Dawei, Hangzhou, China) for the identification and differentiation of members of the genus *Staphylococcus*. Isolates were tested under the same condition for: urease, aesculin hydrolysis, β-galactosidase, arginine dihydrolase and nitrate reduction; acid production from maltose, sucrose, mannose, mannitol, cellobiose, lactose, glycerol, D-melibiose, fructose, sorbitol, and glucuronate; and utilization of pyruvate and acetylglucosamine. The reactions were interpreted by DW-M80 Automated microbial biochemical identification system (Dawei, Hangzhou, China).

Isolates of Gram-positive catalase-positive cocci were further identified by 16S rRNA gene sequencing. Bacterial DNA was extracted and the 16S rRNA gene was amplified using the primers 27F (5′-AGAGTTTGATCCTGGCTCAG-3′) and 1492R (5′-TACGGCTACCTTGTTACGACTT-3′). Sequencing was conducted by Beijing Tsingke Biotechnology Company. The sequences obtained were assembled and compared with those available in GenBank. Alignment with the sequences of representatives of the associated genus *Kocuria* (retrieved on 2 Aug 2020) was conducted using CLUSTAL X (v. 1.8). In order to show the taxonomic position of the isolates in relation to other taxa of the genus *Kocuria*, a phylogenetic tree based on 16S rRNA gene sequence was constructed using neighbor-joining algorithm based on 1,000 bootstrap replications with MEGA X (Hall, 2013). Two published *K. rhizophila* strains and type strains of related species were used as reference stains. *Rothia dentocariosa* strain ATCC 17931 served as the out-group.

### Technological Characterization

#### Salt Tolerance Test

Bacterial cells in an overnight culture were resuspended in saline solution to an OD_600_ of 0.1. The suspension was then mixed with equal volume of nutrient broth containing 10 g/L tryptone, 3 g/L beef extract and different amount of sodium chloride. The final salt concentration of these cultures was 25, 50, 75, and 100 g/L, respectively. The optical density at 600 nm was measured after 24, 48, and 72 h incubation at 37°C. Each sample was done in triplicate.

#### Nitrate Reductase Activity

Nitrate reductase activity was assayed first qualitatively as described previously with modifications (Hu et al., 2018). An overnight culture of each isolate was inoculated into nutrient broth with 0.15% NaNO_3_. After incubation at 37°C for 48 h, 1 mL culture was added to white enamel dish, then 1 mL of solution A (0.8% sulphanilic acid in 5 N acetic acid) and 1 mL of solution B (0.6% *N*-*N*-dimethyl-1-naphthylamine in 5 N acetic acid) were added. The development of red color indicated nitrate reductase activity.

A further quantitative test was conducted as described earlier with modifications (Müller et al., 2016). Cells of an overnight culture were resuspended in nutrient broth containing 0.15% NaNO_3_. This suspension was inoculated in the same fresh broth at a size of 1% and incubated at 37°C for 24 h. Then, 10 μL of this culture, 2,090 μL sterile water, 500 μL solution A, and 500 μL solution B were mixed for 1 min and incubated at room temperature for 15 min. The absorbance was measured in triplicate at 540 nm. A standard curve of nitrite ranging 0.01–2.5 mM was constructed. The production of nitrite was then expressed as mol NO_2_ per 1.0 × 10^7^ CFU.

#### Proteolytic Activity

Proteolytic activity of the isolates was measured by the generation of free amino acids and polypeptides after incubating bacteria with protein extract. Sarcoplasmic protein and myofibrillar protein were extracted according to methods described preciously (Fadda et al., 1999; Basso et al., 2004). The protein obtained was filtered with 0.22 μm filters and the concentration was determined by Bradford method using bovine serum albumin as standard (Bradford, 1976).

Cell suspension (OD_600_ = 0.1) was inoculated at a size of 1% into the sarcoplasmic protein extract solution, which contained 2 g/L sarcoplasmic protein. After incubating at 37°C for 24 h, the culture was centrifuged to remove bacterial cells before the assay. Total free amino acid was measured. The medium without bacterial addition was used as control. Amino acid content was determined by ninhydrin colorimetric method, using glutamic acid as standard. The reaction mixture consisted of 1 mL cell-free culture, 0.5 mL 1/15 M phosphate buffer (pH 8.0) and 0.5 mL 2% ninhydrin solution. The reaction solution was heated in a boiling water bath for 15 min. After cooling and dilution, the absorbance was determined in triplicate at 570 nm.

Proteolytic activity toward sarcoplasmic protein and myofibrillar protein was analyzed by modifying a method reported previously (Cachaldora et al., 2013). Medium containing 1 g/L protein extract and 1 g/L glucose was inoculated with 1% cell suspension (OD_600_ = 1). After incubation at 30°C for 3 days, 80 μL supernatant was mixed with the same volume of 2 × SDS-PAGE loading buffer (Sangon biotech, Shanghai, China), then boiled for 5 min. After centrifuging, the supernatant was loaded on 4–15% SDS-PAGE gel (C621104-0001, Sangon Biotech, Shanghai, China). The molecular weights of the proteolysis products were estimated based on protein standards ranging from 10 to 250 kDa (Bio-rad, 161-0374, CA, United States). Electrophoresis was carried out at 80 V for 8 min and then 120 V for 40 min. Polypeptides were visualized by staining with Coomassie brilliant blue. The resultant polypeptide profile was compared with that of untreated protein extract control. Density and molecular weight of the protein bands were measured using Image Lab software 6.0 (Bio-rad, CA, United States). Two independent experiments were performed for each strain.

#### Lipolytic Activity

Lipolytic activity was analyzed on agar plate modified from a method described earlier (Müller et al., 2016). The medium for lipolytic bacteria screening contained 10 g/L peptone, 3 g/L beef extract, 5 g/L NaCl, 20 g/L agar, and 10 g/L pork oil. A drop of 2 μL cell suspension of overnight culture (OD_600_ = 0.1) was spotted on the plate and then incubated at 37°C for 4 days. The area of clear zone was measured by automatic colony counter (Shineso, Hangzhou, China) every 24 h. Two independent experiments were performed for each strain.

#### Assessment of Antibacterial Potential

Antibacterial activity of isolates K24 and K45 was assessed by a spot-on-a-lawn method (Omar et al., 2006), using *Escherichia coli* ATCC 25922 and *Staphylococcus aureus* ATCC 25923 as indicator organisms. Briefly, overnight *Kocuria* cultures in nutrient broth (NB) were spotted (10 μl) on NB agar. After 24 h of incubation at 37°C, the plates were overlayed with LB agar previously inoculated with indicator strain (10^7^ CFU/mL). After further incubation for 12 h, the antimicrobial activity was determined by observing clear inhibition zones around the spots. Two independent experiments were performed for each strain.

### Safety Characterization

#### Hemolytic Activity

Hemolytic activity was analyzed on blood agar plates (Hopebio, Qingdao, China). A loop of exponential-phase bacteria was streaked on the plates and incubated at 37°C for 24 h. The appearance of a green zone around the colony indicated α-hemolytic activity, whereas a clear halo indicated β-hemolytic activity. No reaction indicated γ-hemolysis or non-hemolysis. Two independent experiments were performed for each strain.

#### Antimicrobial Susceptibility

Due to the fact that there was no current specific standard for antimicrobial application toward *K. rhizophila*, susceptibility of the isolates to 14 clinically used antibiotics, i.e., gentamicin (GEN), amikacin (AMK), tetracycline (TET), ciprofloxacin (CIP), chloramphenicol (CHL), florfenicol (FFC), amoxicillin-clavulanic acid (AMC), ceftazidime (CAZ), cefoxitin (FOX), linezolid (LZD), rifampin (RIF), vancomycin (VAN), erythromycin (ERY) and clindamycin (CLI), was determined in triplicate by the standard Kirby-Bauer disk diffusion method according to CLSI guidelines [Clinical and Laboratory Standards Institute [CLSI], 2020]. *Staphylococcus aureus* ATCC 25923 was used as a control strain.

#### Biogenic Amine Production

The production of biogenic amines by the isolates was analyzed by a semiquantitative method described previously (Joosten and Northolt, 1989). Overnight culture (OD_600_ = 1) was spot inoculated on agar plate supplemented with 2% (w/v) precursor amino acid (ornithine, lysine, tyrosine, or histidine) and then incubated at 37°C for 6 days. The purple circle area, indicating the amino acid decarboxylase activity of an isolate, was determined in triplicate by Icount-20 automatic colony counter (Shineso, Hangzhou, China).

### Genome-Based Assessment of Safety Properties

#### Genome Sequencing and Assembly

Bacterial genomic DNA was extracted using CTAB method (Porebski et al., 1997). The quality and quantity of the DNA extract were examined using a NanoDrop 2000 spectrophotometer (NanoDrop Technologies, DE, United States), Qubit dsDNA HS Assay Kit on a Qubit 3.0 Fluorometer (Life Technologies, CA, United States) and electrophoresis on a 0.8% agarose gel, respectively.

A total of 1 μg DNA per sample was used as input material. Sequencing libraries were generated using the VAHTS Universal DNA Library Prep Kit for MGI (Vazyme, Nanjing, China). Index codes were added to attribute sequences to each sample. Libraries were analyzed for size distribution by Bioanalyzer 2100 system (Agilent Technologies, CA, United States) and quantified using Qubit 3.0 Fluorometer (Life Technologies, CA, United States). Subsequently, sequencing was performed on a MGI-SEQ 2000 platform by Frasergen Bioinformatics Co., Ltd., (Wuhan, China).

Whole genome sequencing was performed with 2 bp × 150 bp read length chemistry. Quality control of the paired-end data was performed using “FastQC” (Andrews, 2010). Good quality filtered reads were assembled into scaffolds using “SOAPdenovo” v2.04 (Li et al., 2017). A final gap-filling step was performed using “GapCloser” v1.12 (Hanna et al., 2017) to generate a draft genome.

Gene prediction was performed using Glimmer (v3.02) (Delcher et al., 2007). The annotation of genes was achieved by diamond (v0.9.12.113) with an E-value threshold of 1e-5 against the databases of the NCBI Non-Redundant protein database (NR), Swiss-Prot, Clusters of Orthologous Groups (COG), Kyoto Encyclopedia of Genes and Genomes (KEGG), and Gene Ontology (GO). The match with the highest score was considered to be the final annotation of one specific gene.

The resulting sequences of *K. rhizophila* K24 and K45 were submitted to GenBank under accession numbers JAEUXN000000000 and JAEUXO000000000, respectively.

#### Analysis of Pathogenicity Genes and Antimicrobial Resistance Genes

In order to confirm the safety of the *K. rhizophila* isolates, their draft genomes were screened for the presence of virulence genes using BLAST against full dataset of DNA sequences from virulence factor database (VFDB^1^). Acquired antimicrobial resistance genes were identified by ResFinder 4.1^2^ with default settings. Predicted protein sequences from the genomes were also aligned with the comprehensive antibiotic resistance database (CARD, v3.0.1) using diamond with an E-value threshold of 1e-5. The database queries were conducted in December 2020.

#### Analysis of Decarboxylase Genes for Biogenic Amine Production

The assembled gene sequences of *K. rhizophila* K24 and K45 were each used to build database using the makeblastdb command in BLAST 2.6.0 + toolkit. The reference gene sequences encoding for tyrosine decarboxylase, histidine decarboxylase, lysine decarboxylase, and ornithine decarboxylase were retrieved from NCBI Nucleotide database (on 16 March 2021). The corresponding gene sequences of phyla *Actinobacteria* and *Firmicutes* were used as input sequences and searched against the aforementioned K24 and K45 database using Nucleotide-Nucleotide BLAST 2.6.0+. Genes were considered to be present in the genome of the *K. rhizophila* isolates if a hit with ≥ 80% sequence identity over ≥ 50% of the length of query gene was found.

### Statistical Analysis

Statistical significance was determined by one-way analysis of variance (ANOVA) with Tukey test (*p* < 0.05) using Origin 8.6 (OriginLab Inc., Northampton, MA, United States).

## Results and Discussion

### Isolation and Identification of Proteolytic Bacteria

Nuodeng ham samples were collected from two local producers. A total of 65 colonies on different culture media were chosen and isolated. Out of them, 14 isolates showed large clear zones after incubating on litmus milk agar with the radius of a clear zone 1.5–3.6 times that of the colony, indicating proteolytic activity. Nine proteolytic isolates were found to be Gram-positive catalase-positive cocci, which were subsequently identified by 16S rRNA gene sequencing. One isolate was found related to *Dermacoccus nishinomiyaensis*, whereas the other eight, derived from both local producers, were found closely related to *K. rhizophila*.

The eight *Kocuria* strains were firstly subjected to biochemical characterization (Table 1). The results demonstrated that although the isolates reacted similarly in most tests, they differed from each other in terms of the reaction profile. Except for urease reaction, the biochemical characteristics observed for the isolates were consistent with those reported for *K. rhizophila* DSM 11926^T^ (Kovács et al., 1999; Braun et al., 2019). The deviation may result from different conditions used for characterization.

**TABLE 1:** Biochemical characteristics of eight *Kocuria* isolates.

Characteristic	K18	K19	K22	K24	K45	K46	K56	K60
Urease	+	+	+	+	+	+	+	+
Aesculin hydrolysis	–	–	–	–	–	–	–	–
β-Galactosidase	–	–	–	–	–	–	–	–
Arginine dihydrolase	–	–	–	–	–	–	–	–
Nitrate reduction	–	–	–	+	+	+	+	–
Maltose	+	+	+	+	+	+	–	+
Sucrose	+	+	+	+	+	+	–	+
Mannose	+	–	+	–	–	–	–	–
Mannitol	–	–	–	–	–	–	–	–
Cellobiose	–	–	–	–	–	–	–	–
Lactose	–	–	–	–	–	–	–	–
Glycerol	+	+	+	+	–	+	+	+
D-Melibiose	+	+	+	+	+	+	–	+
Fructose	+	+	+	+	+	+	+	+
Sorbitol	–	–	–	–	–	–	–	–
Glucuronate	–	–	–	–	–	–	–	–
Pyruvate	+	+	+	+	+	+	+	-
Acetylglucosamine	–	–	+	+	–	–	–	–
Pigment color	yellow	yellow	yellow*	yellow	yellow*	orange	orange	yellow

*Colony color changed from yellow to orange after 5 days incubation at 37°C on MSA agar.

*+, Positive; –, negative.*

**FIGURE 1:**
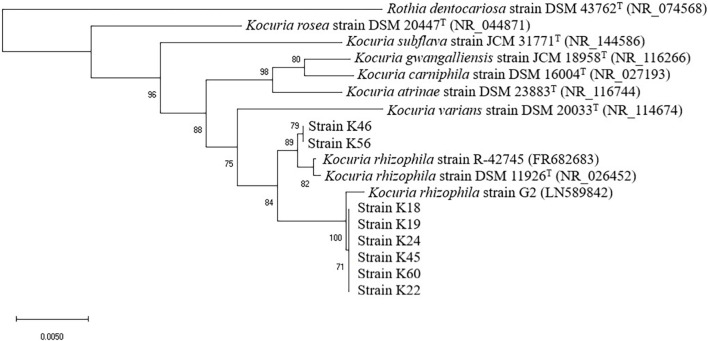
Neighbor-joining phylogenetic tree of eight isolates from Nuodeng hams and reference strains of the genus *Kocuria* constructed using their 16S rRNA gene sequences. The numbers at the nodes are bootstrap probabilities in percent. The scale bar indicates the number of substitutions per nucleotide position.

As revealed by the phylogenetic tree based on 16S rRNA gene sequence similarity (Figure 1), the *Kocuria* isolates were generally separated into two clusters with other *Kocuria* strains. Isolates K46 and K56 clustered together and showed high homology, which was consistent with their distinct orange colony color. These two isolates formed a clade with *K. rhizophila* DSM 11926^T^ and *K. rhizophila* strain R-42745. Isolate K46 showed 99.28% identity with the type strain, and K56 showed 99.29% identity. The other six isolates (K18, K19, K22, K24, K45, and K60) clustered with *K. rhizophila* strain G2. They had identity to *K. rhizophila* DSM 11926^T^ at 99.29, 99.29, 99.07, 99.24, 98.85, and 99.06%, respectively. Among these six isolates, colony color of K22 and K45 changed from yellow to orange during incubation, which was different from other four yellow isolates.

### Technological Characterization

Technological evaluation of *K. rhizophila* isolates was carried out *in vitro* in view of their application as meat starter cultures. As members of CNC, all eight *K. rhizophila* isolates were able to produce catalase, as shown by a large amount of bubbles formed once in contact with hydrogen peroxide. Catalase activity of meat starter culture can help prevent excessive lipid oxidation and unwanted discoloration in fermented meats (Talon et al., 1999).

#### Salt Tolerance

The halotolerant character of meat starter would allow them to remain active in meat curing process as salt content increases. All eight *K. rhizophila* isolates grew well in 50 g/L salt medium, however, when salt concentration increased, the isolates showed varying salt tolerance. Under a salt concentration of 75 g/L, the growth of K18, K22, K46 decreased the most, while the growth of other isolates decreased to a less extent, except K24, whose growth even increased slightly compared with that under 50 g/L salt concentration (data not shown). In 100 g/L salt medium, isolate K24 showed obvious growth after 72 h, while isolates K46 and K56 showed the least growth (Table 2). In an earlier study, an *K. rhizophila* isolate showed higher salt tolerance than strains of other *Kocuria* species, which grew under salt concentration as high as 15% (Kovács et al., 1999).

**TABLE 2:** Test results of eight *Kocuria rhizophila* isolates for salt tolerance, nitrate reductase activity, and proteolytic activity.

Samples	OD_600_ after 72 h in medium with 100 g/L salt	Nitrite formation in nitrate reductase activity test (mol NaNO_2_ per 1 × 10^7^ CFU)	Total free amino acid (g/L) generated from sarcoplasmic protein extract medium
K18	0.11 ± 0.00^e^	–	1.55 ± 0.03^bc^
K19	0.16 ± 0.00^b^	–	1.73 ± 0.15^a^
K22	0.13 ± 0.00^d^	–	1.53 ± 0.01^c^
K24	0.25 ± 0.01^a^	0.47 ± 0.01^a^	1.51 ± 0.04^c^
K45	0.14 ± 0.01^c^	0.46 ± 0.08^a^	1.73 ± 0.02^a^
K46	0.08 ± 0.01^f^	0.28 ± 0.10^b^	1.57 ± 0.02^bc^
K56	0.09 ± 0.00^f^	0.27 ± 0.09^b^	1.59 ± 0.02^bc^
K60	0.13 ± 0.00^d^	–	1.65 ± 0.01^ab^
Control	0.05 ± 0.00^g^	–	1.14 ± 0.06^d^

*^a–g^Different letters in the same column represent significant differences (p < 0.05).*

#### Nitrate Reductase Activity

CNC with high nitrate reductase activity are expected to help develop characteristic cured meat color and avoid undesired flavor (Landeta et al., 2013). All eight *K. rhizophila* isolates showed nitrate reductase activity by the appearance of red color in the qualitative test. In the further quantitative test, isolates K24 and K45 exhibited the highest nitrate reductase activity with nitrite accumulation of 0.47 and 0.46 mol NaNO_2_ per 1 × 10^7^ CFU (Table 2), which is comparable to that of *Staphylococcus carnosus* starter cultures (Müller et al., 2016). Isolates K46 and K56 produced lower amount of nitrite, while other isolates didn’t show nitrite production within the incubation time in the quantitative test.

#### Proteolytic Activity

Isolates were cultured in sarcoplasmic protein extract medium for their ability of releasing free amino acid. All isolates generated significantly higher amount of total free amino acid than the control (Table 2). The release of amino acid in the uninoculated control might be due to the activity of endogenous enzymes in protein extract.

**FIGURE 2:**
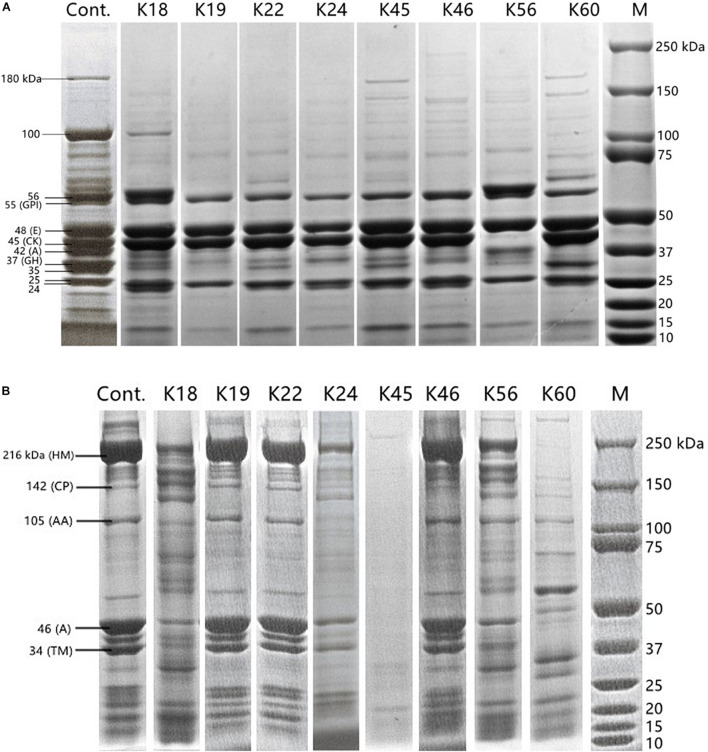
SDS-PAGE polypeptide profile of sarcoplasmic protein **(A)** and myofibrillar protein **(B)** inoculated with the *Kocuria* isolates and uninoculated control. Labels in panel **(A)**: GPI, glucose phosphate isomerase; E, enolase; CK, creatine phosphate kinase; A, aldolase; GH, glyceraldehyde phosphate dehydrogenase. Labels in **(B)**: HM, H-meromyosin; CP, C protein; AA, α-actinin; A, actin; TM, tropomyosin.

Changes in polypeptide profile of sarcoplasmic protein and myofibrillar protein by the hydrolysis of the *Kocuria* isolates were monitored (Figure 2). The profile of sarcoplasmic protein extract is shown in Figure 2A. According to their molecular weight, bands can be glucose phosphate isomerase (55 kDa), enolase (48 kDa), creatine phosphate kinase (45 kDa), aldolase (42 kDa) and glyceraldehyde phosphate dehydrogenase (37 kDa) (Cachaldora et al., 2013). The bands of 180 kDa and 100 kDa were degraded by most isolates. The proteins of 55 kDa, 48 kDa, and 25 kDa remained intact after incubation with the isolates. The band of 45 kDa was degraded only by K56. Except for K18 and K56, aldolase band (42 kDa) was hydrolyzed. K19, K56 and K60 degraded the band of 37 kDa to a higher degree than other isolates. K19 and K56 also showed activity toward the band of 24 kDa.

SDS-PAGE profile of myofibrillar protein extract (Figure 2B) contained bands of 216 kDa (H-meromyosin), 142 kDa (C protein), 105 kDa (α-actinin), 46 kDa (actin), 34 kDa (tropomyosin) (Martìn et al., 2002). K45 showed the highest activity on myofibrillar protein, followed by K24 and K60. Almost no hydrolysis of myofibrillar protein was caused by K19, K22 and K46. Zeng et al. (2017) reported that *Staphylococcus* spp. isolated from fermented sausages were more proteolytic for myofibrillar proteins than for sarcoplasmic proteins. Compared to the proteolysis product profile of coagulase-negative staphylococci (Cachaldora et al., 2013), the most commonly used meat starters, *K. rhizophila* may supplement their proteolytic pathway and lead to a more diverse profile of flavor compounds.

#### Lipolytic Activity

Lipolytic CNC play a role in releasing fatty acid precursors and improving meat sensory profile (Sanchez Mainar et al., 2017). However, for the eight *Kocuria* isolates, no clear zone formation was observed on the test agar plate containing pork oil, indicating their weak lipolytic activity. Similarly, no lipase activity was observed for a *K. rhizophila* isolate from extra virgin olive oil (Fancello et al., 2020). A sausage-derived *K. varians* isolate was found negative for lipolytic activity (Martin et al., 2006). Coagulase-negative staphylococci from sausages showed a higher percentage of lipolytic isolates (Martin et al., 2006; Sun et al., 2019).

#### Antibacterial Activity

Two isolates K24 and K45 were selected for antibacterial activity test. Neither isolate formed inhibition zones on agar containing *E. coli* and *S. aureus* indicator organism. *K. varians* isolates from raw salami fermentation were reported to produce a bacteriocin named variacin, which inhibited a wide range of Gram-positive bacteria, including *Listeria* spp., *Staphylococcus* spp. and bacilli, but inactive toward Gram-negative bacteria (Pridmore et al., 1996).

#### Safety Characterization

*Kocuria rhizophila* has been isolated from a variety of sources, e.g., soil, freshwater, foods, and healthy human gut (Anang et al., 2006; El-Baradei et al., 2007; Ghattargi et al., 2018). It has also been used as standard quality control strain for antimicrobial susceptibility testing. However, *K. rhizophila* has been rarely reported as opportunistic pathogen in immunocompromised patients and to cause bacteremia due to its affinity to catheter (Moissenet et al., 2012; Kogure et al., 2014). It is therefore important to evaluate the potential risks of *K. rhizophila* isolates to human health in the selection of novel starter cultures.

#### Hemolytic Test

Hemolytic activity associated with bacterial virulence was evaluated, and all eight *K. rhizophila* isolates showed Gamma hemolysis, indicating negative for hemolytic activity. Marty et al. (2012) evaluated the potential of 5 *S. xylosus* and 17 *S. equorum* isolates as meat starter culture, and phenotypic hemolytic activity was found for 3 *S. xylosus* and 15 *S. equorum* isolates. In another study, 77 CNC isolates from raw milk and cheese were tested for hemolytic activity, and 24 isolates were found positive, with 17 showing β-hemolysis and 7 α-hemolysis (Ruaro et al., 2013).

#### Antimicrobial Susceptibility Test

Due to the lack of CLSI interpretive criteria for *Kocuria*, the antimicrobial susceptibility test results of *Kocuria* isolates were interpreted by referring to *Staphylococcus*. All eight strains were susceptible to GEN (inhibition zone diameters varied between 22 and 29 mm), AMK (23–27 mm), TET (22–27 mm), CIP (21–30 mm), CHL (26–31 mm), FFC (26–30 mm), AMC (27–34 mm), CAZ (24–29 mm), FOX (20–31 mm), LZD (28–31 mm), RIF (24–31 mm), VAN (17–28 mm) and ERY (23–30 mm). Isolate K22 was resistant to CLI (10 mm), while the other seven isolates were susceptible (24–30 mm). Two *K. rhizophila* isolates from diseased fish were reported sensitive to the tetracyclines, β-lactams, macrolide, and amphenicol tested, as shown by a large zone of growth inhibition (Pȩkala et al., 2018). In another study of prevalence of antibiotic resistance in coagulase-negative *Staphylococcus* isolates from spontaneously fermented meat products, 49% isolates among six species showed resistance to at least one antibiotic and the resistance was found to be species dependent (Marty et al., 2012). Low prevalence of phenotypic antibiotic resistance in *K. rhizophila* isolates suggested an advantage of this species as a starter candidate.

#### Biogenic Amine Production Assay

The biogenic amine productivity of eight *K. rhizophila* isolates was examined on agar medium supplemented with ornithine, lysine, tyrosine, or histidine after 6 days incubation. Isolate K19 developed a purple color area around its colony on ornithine, lysine, and tyrosine agar medium with an average area of 20.54, 16.47, and 6.02 cm^2^, respectively, thus positive for putrescine, cadaverine and tyramine production. Isolate K22 showed 7.07 cm^2^ purple area on ornithine agar medium. The decarboxylase activity was not detected for the rest (6/8) of the isolates. The production of the amines thus appeared to be strain-specific and not common in *K. rhizophila* isolates.

According to the present study, none *K. rhizophila* isolates produced histamine, the most toxic amine in food. In an earlier study, 29 *K. varians* isolates were tested for histamine and tyramine production on agar plates, and all of them decarboxylated histidine and only four failed to decarboxylate tyrosine (Fadda et al., 2001). The concentration of precursor amino acid used in this earlier study was 1,000-fold lower than that used here, and it was thus unlikely that our method failed to detect biogenic amine production by the *K. rhizophila* isolates. Jančová et al. (2020) analyzed the ability of two *K. rhizophila* and one *K. varians* isolates from dairy wastewater to produce eight biogenic amines. Except for spermidine, the production of other biogenic amines was detected, with that of putrescine, cadaverine and tyramine being the most significant (Jančová et al., 2020). It should be noted that only the production of the biogenic amines of most concern was examined in the present study. The isolates’ potential to reduce the risk of biogenic amine contamination remains to be evaluated.

### Genome-Based Assessment of Safety Properties

*Kocuria rhizophila* K24 and K45 were selected for their overall performance on salt tolerance, nitrate reductase and proteolytic activity, as well as the absence of antimicrobial resistance and amino acid decarboxylase activity. Genome sequences of K24 and K45 were obtained to confirm that the isolates are safe for use as starter cultures in meat fermentation. No plasmid was detected in either genome according to the plasmid annotation results. After assembling, K24 and K45 were shown to have a genome size of 2.69 and 2.74 Mb, and G + C content of 71.34 and 71.39%, respectively. A total of 2,404 and 2,459 genes were predicted to be present in isolates K24 and K45, respectively. These genes were classified according to their functions, and the genes involved in amino acid transport and metabolism comprised the largest gene category (Supplementary Figure 1), suggesting the capacity of *K. rhizophila* in converting amino acids into flavor compounds. The flavor-affecting metabolic activity of *K. rhizophila* may contribute to flavor development of artisanal Nuodeng ham. The complete genome sequences of *K. rhizophila* of cured meat origin would facilitate further comparative and functional genomic analysis of this species.

No virulence gene in the genome sequences of the two isolates was found by searching the VFDB. A search against the ResFinder and CARD databases identified no antimicrobial resistance gene in the two isolates. The genes encoding for tyrosine decarboxylase, histidine decarboxylase, lysine decarboxylase, and ornithine decarboxylase were also found missing from the two *K. rhizophila* isolates, in line with the observation of their non-producing phenotype for the biogenic amines. Genome sequencing of food-derived *S. carnosus*, *S. equorum*, *S. succinus*, and *S. xylosus* isolates showed that they all carried lysine decarboxylase-encoding genes required for cadaverine production, and none carried genes involved in histamine and tyramine production (Heo et al., 2020).

## Conclusion

In the present study of native bacteria from artisanal Nuodeng ham in China, a high proportion of isolates (8/14 isolates) with high proteolytic activity were affiliated with *Kocuria rhizophila*. The potential of *K. rhizophila* isolates as a new source of CNC starter cultures was evaluated for the first time. Among the eight isolates, differences in physiological and biochemical properties were observed. Phylogenetic diversity of the isolates was found corresponding to their diverse colony morphology. *In vitro* screening was conducted based on technologically relevant properties, such as salt tolerance, nitrate reduction and proteolytic activity. Although none isolates tested showed lipolytic activity or antibacterial activity on agar plates, the results added to current knowledge on this species. Moreover, *in vitro* and *in silico* safety assessment of *K. rhizophila* isolates was conducted. The isolates were all negative for hemolytic activity and sensitive to the 14 antimicrobials tested, except for one isolate to clindamycin. The majority (6/8) of them did not show decarboxylase activity toward ornithine, lysine, tyrosine, and histidine for biogenic amine production. Genome analysis of isolates K24 and K45 found no safety concern regarding to pathogenicity, antimicrobial resistance and common biogenic amine production. The desirable functional and safety properties of the selected *K. rhizophila* isolates make them promising starter candidates for meat fermentation. Further study needs to assess *in vivo* effects of the selected *K. rhizophila* isolate on sensory qualities of cured meats.

## Data Availability Statement

The datasets presented in this study can be found in online repositories. The names of the repository/repositories and accession number(s) can be found below: https://www.ncbi.nlm.nih.gov/genbank/, JAEUXN000000000; https://www.ncbi.nlm.nih.gov/genbank/, JAEUXO000000000.

## Ethics Statement

The animal study was reviewed and approved by the Ethics Committee of Institute of Agro-Products Processing, Yunnan Academy of Agricultural Sciences.

## Author Contributions

QS and HL contributed to conception and design of the study. XW, ZJ, and BL performed the experiments. CL and HW contributed to data analysis. QS and XW prepared the manuscript. All authors contributed to manuscript revision, read, and approved the submitted version.

## Conflict of Interest

The authors declare that the research was conducted in the absence of any commercial or financial relationships that could be construed as a potential conflict of interest.

## Publisher’s Note

All claims expressed in this article are solely those of the authors and do not necessarily represent those of their affiliated organizations, or those of the publisher, the editors and the reviewers. Any product that may be evaluated in this article, or claim that may be made by its manufacturer, is not guaranteed or endorsed by the publisher.

## Footnotes

1
http://www.mgc.ac.cn/VFs/blast/

2
https://cge.cbs.dtu.dk/services/ResFinder/
