# Supplementary material for: Technological and Safety Characterization of Kocuria rhizophila Isolates From Traditional Ethnic Dry-Cured Ham of Nuodeng, Southwest China
Source: Front Microbiol. 2021 Nov 15;12:761019. doi: 10.3389/fmicb.2021.761019 (PMC8634685; doi:10.3389/fmicb.2021.761019)
Supplement: Supplementary file 1 [file Table_1.DOCX]

A

B

Fig. S1 The COG classification graphs of *Kocuria rhizophila* isolate K24 (A) and isolate K45 (B).
